# Decreasing trends, and geographical variation in outpatient antibiotic use: a population-based study in Central Denmark

**DOI:** 10.1186/s12879-019-3964-9

**Published:** 2019-04-24

**Authors:** Pia Kjær Kristensen, Søren Paaske Johnsen, Reimar Wernich Thomsen

**Affiliations:** 1 Department of Clinical Epidemiology, Aarhus University Hospital, Olof Palmes Allé 43-45, DK-8200 Aarhus N, Denmark; 2 Department of Orthopedic Surgery, Regional Hospital Horsens, Sundvej 30, DK-8700 Horsens, Denmark; 3 Department of Clinical Medicine, Aalborg University, Sdr. Skovvej 15, DK-9000 Aalborg, Denmark

**Keywords:** Antibiotics, Drug utilization, Outpatient prescriptions, Trends

## Abstract

**Background:**

Antimicrobial overuse and misuse of broad-spectrum antibiotics increases the risk for antimicrobial resistance. Investigating unwarranted variation in antibiotic prescription has therefore gained global priority.

**Methods:**

We examined recent time trends in the utilization of narrow- and broad-spectrum antibiotics as well as the variation in antibiotic use by sex, age, and municipality of residence. Complete individual-level data on all redeemed out-of hospital prescriptions for antibiotics in the entire adult population of Central Denmark (1.3 million inhabitants) was obtained for the period 2006–2015.

**Results:**

Following an initial increase of 2% between 2006 and 2011, the overall rate of redeemed prescriptions for antibiotics per 1000 person years declined by 17% between 2011 and 2015. Among persons aged over 65 years, the decline in use began later (from 2013) and was less pronounced. Antibiotic use in 2015 remained substantially higher among females (289/1000 person-years) vs. males (182/1000 person-years) and among the very old (520/1000 person-years in >85y old) vs. middle-aged (204/1000 person-years in 45-65y old). A decreasing trend in antibiotic use over time was observed in all municipalities, mainly due to a decrease in narrow-spectrum antibiotics. However, a striking and unexplained 1.6-fold geographical variation in antibiotic use, including tetracyclines, macrolides and fluoroquinolones remained in 2015. Of concern, among females aged ≥65 years and males aged ≥85 years, a continuous increasing trend in broad-spectrum antibiotic use was observed.

**Conclusions:**

Antibiotic use has decreased almost 20% in Central Denmark after 2011, possibly related to a nationwide antibiotic stewardship program in Denmark. However, substantial geographical variation in antibiotic prescription remains and the use of broad-spectrum antibiotics has increased in adults of older age. Continuous focus on avoiding unnecessary use of broad-spectrum antibiotics is requested.

**Electronic supplementary material:**

The online version of this article (10.1186/s12879-019-3964-9) contains supplementary material, which is available to authorized users.

## Background

Healthcare systems worldwide face the threat of antibiotic resistance, rendering patients at risk of ineffective treatment and increasing healthcare costs. Antimicrobial overuse and misuse is an important risk factor for antimicrobial resistance and appropriate antibiotic prescribing behavior is consequently important to ensure patient safety and quality of care [1–4]. However, real world antibiotic prescribing behavior does not always comply with clinical guidelines recommendations [5] and investigating unwarranted variation in antibiotic prescription has therefore gained priority in both the European Union and the United States [6–11].

Studies on antibiotic utilization in the European countries have shown increasing overall antibiotic prescribing rates during the early 2000s, while the prescribing rates apparently decreased in the United States from 2006 to 2010 [7, 8, 11]. Newer data on outpatient antibiotic use are scarce. Furthermore, studies have shown up to four-fold variation in antibiotic use within the European Union [6, 8, 10, 11], both between countries and between geographical regions in individual countries [12–15]. Striking variation has also been observed in the utilization of specific antibiotics, including penicillins with extended spectrum, macrolides and quinolones [11]. The increased use of these subgroups is particular problematic as they are associated with growing resistance problem [16, 17]. Although the Danish prescribing rates for antibiotics are low compared with the US and many other European countries [6], antibiotic use in Denmark increased from 1997 to 2007 [8]. Furthermore, a remarkably high use was recently observed among people aged over 65 years in Denmark compared to the Italy, Germany, the Netherlands and United Kingdom [8].

In 2012, after the total consumption of antibiotics in humans had steadily increased throughout the 2000s in Denmark, the national Danish Health Authorities published “Guidelines on prescribing antibiotics for physicians and others in Denmark” [18]. The nationwide initiative focused on reducing unnecessary antibiotic use including the use of critically important antibiotics such as carbapenems, fluoroquinolones and cephalosporins in an outpatient setting [19]. Moreover, a targeted campaign towards the population called “antibiotics or not” with information on adverse effects from antibiotics was initiated. We hypothesize that the national stewardship programs may have contributed to a more rational use of antibiotics in our region, Central Denmark. Correspondingly, annual reports from the Danish Ministry of Health (Statens Serum Institut) have indicated a recent decline in the overall use of antibiotics in primary health care in Denmark from 2011 to 2017, however, these reports do not include detailed analyses regarding patient characteristics and geographics [20]. It is therefore unknown whether the national stewardship programs also contributed to appropriate antibiotic use within different groups of patients and within different municipalities in Central Denmark.

Unlike most countries, the availability of population-based community pharmacy prescription data at the individual person level in Denmark makes it possible to investigate not only time trends in overall antibiotic utilization, but also use of individual antibiotic prescriptions, including their dose and treatment duration, by patient sex, age group, and geographical residence. We therefore conducted a population-based drug utilization study to examine changes in antibiotic prescribing within the last decade including particular types of antibiotics in relation to age, sex and municipality.

## Methods

We conducted a population-based cohort study among all inhabitants above 15 years living in Central Denmark Region from 2006 to 2015. The Central Denmark Region is one of five regions in Denmark, which covers both urban and rural areas. The region has 1.3 million inhabitants, corresponding to 23% of the Danish population [21]. The population of the region is representative for the entire country with respect to healthcare usage, medication use, demographic, as well as socioeconomic characteristics [22]. The Danish National Health Service provides universal healthcare financed by tax, which guarantee unfettered access to healthcare services and partial reimbursement for prescribed drugs. All Danish citizens are provided with a unique personal registry number, which enable unambiguous linkage of all registries at the individual level.

### Data sources

The Danish Civil Registration System was used to identify cohort of inhabitants above 15 years living in Central Denmark Region. The Danish Civil Registration System contains electronic records on vital status (date of birth, emigration and death), and place of residence for the entire Danish population since 1968, which allow us to keep track of the inhabitants included. The registry is updated daily [23].

The Danish National Health Service Prescription Database was used to obtain data on antibiotic use. The database contains complete data on all reimbursed prescription medications dispensed from community pharmacies [24]. The drugs in the Danish National Health Service Prescription Database are coded according to the Anatomical Therapeutic Chemical (ATC) classification system. The database contains information on prescriptions and dispensing including Defined Daily Doses (DDD) (WHO, version 2011) [25].

### Antibiotic utilization

Use of antibiotics was defined, as antibiotic for systemic use including all drugs within Anatomical Therapeutic Chemical (ATC)-group J01. Data was aggregated at the level of active chemical substance using ATC classification. We dived the use of antibiotics into tetracyclines (J01A), amphenicols (J01B), beta-lactam antibacterial penicillins (J01C), cephalosporins (J01D), trimethoprim and sulphonamides (J01E), macrolides and lincosamides (J01F), aminoglycosides (J01G), quinolones (J01 M) and other antibacterials (J01X).

### Statistical analysis

We calculated the annual prescribing rates of antibiotic use per 1000 person-years calculated as the number of inhabitants who filled at least one antibiotic prescription divided by the total cumulated person time in the observation year and multiplied by 1000. We presented the prescribing rates of antibiotic use stratified for sex and age group (15–40 years, 40–65 years, 65–85 years, > 85 years). We also estimated the volume of antibiotic use as Defined Daily Dose (DDD) per 1000 inhabitants per day as overall and stratified for sex and age. Furthermore, we investigated temporal trends 2006–2016 in both prescribing rates and volume of antibiotic use. To investigate intra-regional differences in the use of antibiotics, we estimated sex and age standardized prescribing rates of antibiotic use, and age and sex standardized volume of antibiotic use (DDD per 1000 inhabitants per day) by each municipality in the region stratified by year. The standardization was performed in relation to data from the entire Central Denmark Region in 2006.

We repeated the analyses for narrow- and broad-spectrum antibiotics separately. Narrow spectrum antibiotics were defined as beta-lactamase sensitive penicillins (J01 CE), beta-lactamase resistant penicillins (J01CF), first-generation cephalosporins (J01DB), and macrolides (J01FA01). Broad-spectrum antibiotics included combinations of penicillins including beta-lactamase inhibitors (J01CR), penicillins with extended spectrum (J01CA), second-generation cephalosporins (J01 DC), third-generation cephalosporins (J01DD), macrolides, lincosamides and streptogramins (J01F). We also investigated the use of each of the following classes of antibiotics: Tetracyclines (J01AA), penicillins with extended spectrum (J01CA), beta-lactamase sensitive penicillins (J01 CE), beta-lactamase resistant penicillins (J01CF), combinations of penicillins including beta-lactamase inhibitors (J01CR), trimethoprim and sulphonamides (J01E), macrolides, lincosamides and streptogramins (J01F), fluoroquinolones (J01MA), metronidazol (G01AF01, J01XD01, P01AB01) and other antibacterials (J01X). We calculated both antibiotic prescribing rates and volume of antibiotic use (DDD per 1000 inhabitants per day) of the subgroups of antibiotics.

Additionally, we examined the subgroups of antibiotics within the municipalities and calculated the mean duration of antibiotic use per prescription by adding the total days of antibiotic exposure (DDD*packsize) divided by the total number of prescriptions retrieved for the calendar year 2015.

All analyses were performed using SAS version 9.4.

## Results

**Fig. 1:**
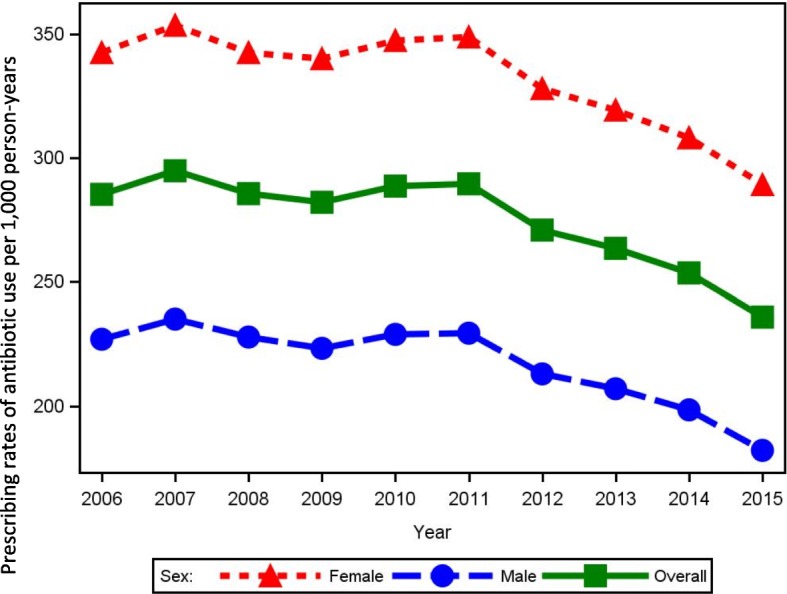
Prescribing rates of antibiotic use per 1000 person-years 2006 to 2015, overall and stratified by sex

**Fig. 2:**
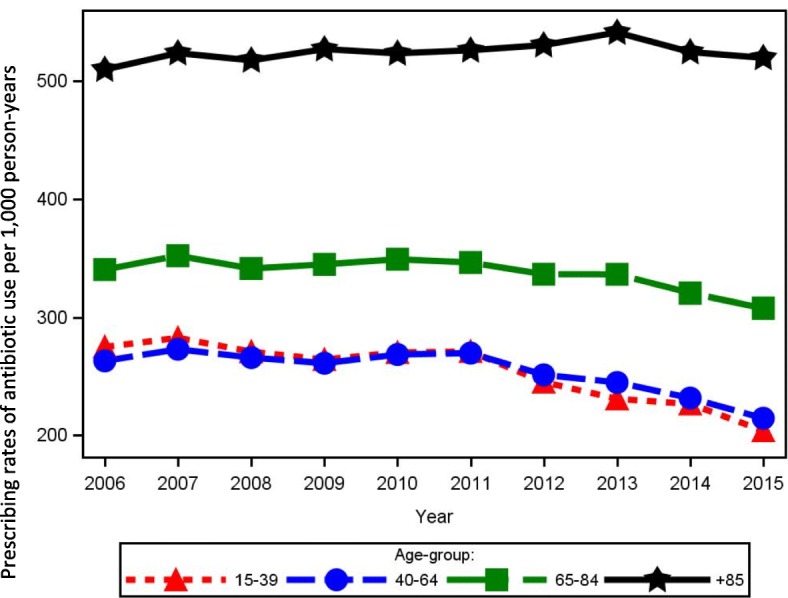
Prescribing rates of antibiotic use per 1000 person-years 2006 to 2015, stratified by age group

**Fig. 3:**
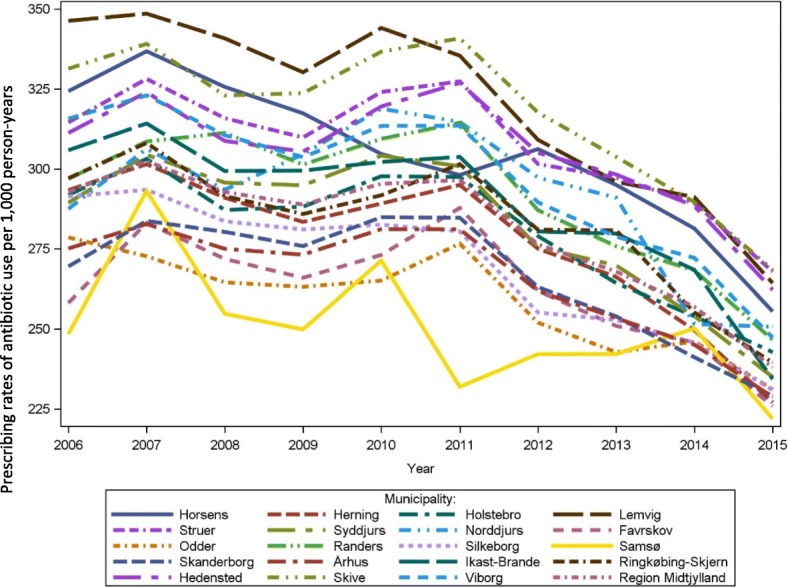
Age and sex standardized prescribing rates of antibiotic use per 1000 person-years by municipalities. Age and sex standardized prevalence of antibiotic use per 1000 person-years 2006 to 2015: variation according to the municipalities in Central Denmark Region

The overall age and sex standardized prescribing rates of antibiotic use in Central Denmark Region increased slightly from 285/1000 person-years in 2006 to 290/1000 person-years in 2011 after which the antibiotic prescribing rates decreased to 236/1000 person-years in 2015. Figure 1 shows the overall prescribing rates of antibiotic use per 1000 person-years from 2006 to 2015 and stratified for females and males. The decrease from year 2011 and forward was found among both sexes (Fig. 1). The lower overall use of antibiotics over time appeared primarily to be related to a decreasing use in the youngest age groups (Fig. 2). In contrast, the prescribing rates of antibiotic use among adults above 85 years increased from 510/1000 person-years in 2006 to 541/1000 person-years in 2013. From 2013 through 2015 there was a small decrease in prescribing rates towards 520/1000 person-years for adults above 85 years. However, a more than 2-fold variation between the youngest and oldest age groups remained in the calendar year 2015 (Fig. 2). The decrease in antibiotic use over time was present in all municipalities in the region, but remarkable geographical variation in sex and age standardized antibiotic utilization was still seen in the municipalities, especially in the period before 2011 (Fig. 3).

When estimating the volume of antibiotic, we saw a similar picture with decrease in overall volume of antibiotics from 5589 DDD/1000 persons in 2011 to 4709 DDD/1000 persons in 2015 (Additional file 1: Table S2). The decrease in antibiotic volume was seen for both females and males (Additional file 1: Table S2). There was an 8-fold variation in antibiotic volume used across age groups, from 2741 DDD/1000 person-years for the youngest age group to 22,332 DDD/1000 person-years for adults above 85 years (Additional file 1: Table S2).

**Fig. 4:**
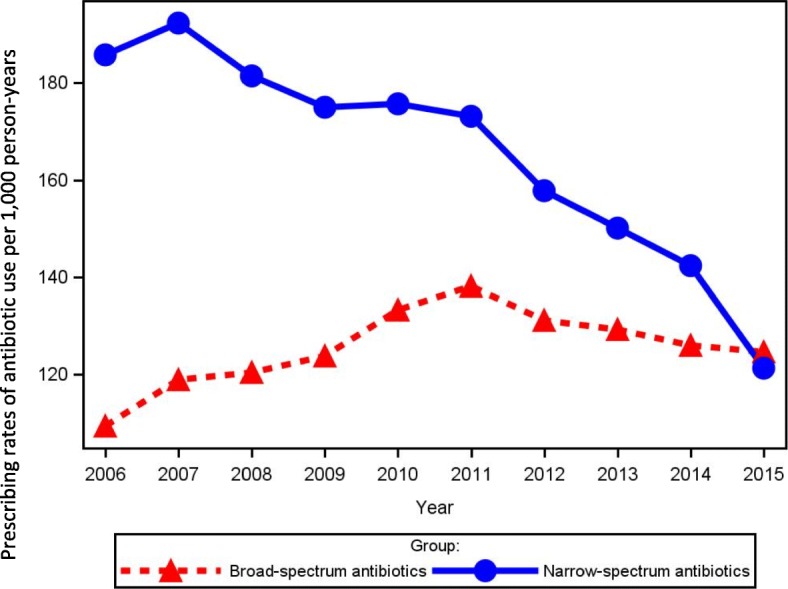
Prescribing rates of broad- and narrow-spectrum antibiotic use per 1000 person-years 2006 to 2015

**Fig. 5:**
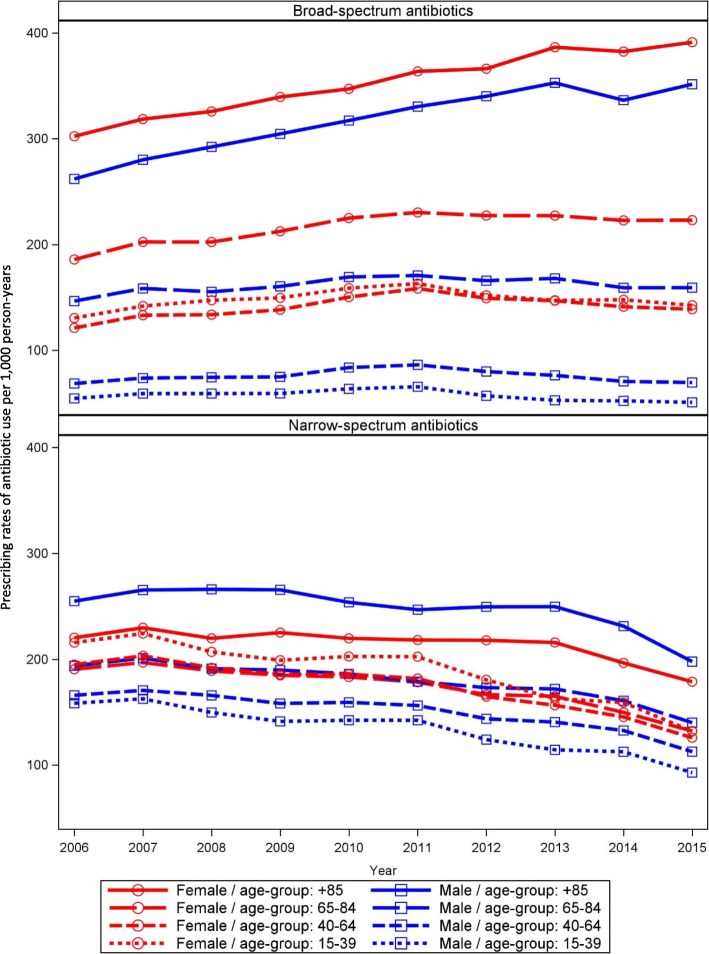
Prescribing rates of broad- and narrow-spectrum antibiotic use per 1000 person-years by sex and age group

The steady decrease in prescribing rates of overall antibiotic use since 2011 was mainly due to a decrease in the prescribing rates of narrow-spectrum antibiotic use (Fig. 4). The decrease in prescribing rates of narrow-spectrum antibiotics started in 2007, and was seen for both females and males as well as for all age groups, but particularly for females aged 15–39 years (Fig. 5). The prescribing rates of broad-spectrum antibiotic increased from 109/1000 persons in 2006 to 138/1000 persons in 2011, after which a small decrease to 125/1000 persons in 2015 was observed. However, the prescribing rates for broad-spectrum antibiotic increased for females above 65 years and males above 85 years (Fig. 5). The small decrease in overall broad-spectrum antibiotic from 2011 was apparently confined to males and females below 40 years. In addition, the prescribing rates of broad-spectrum antibiotic use in 2015 had become higher than that of narrow-spectrum antibiotics (Fig. 4). A similar picture was seen for the volume of narrow- and broad-spectrum antibiotics (Additional file 1: Table S3).

**Fig. 6:**
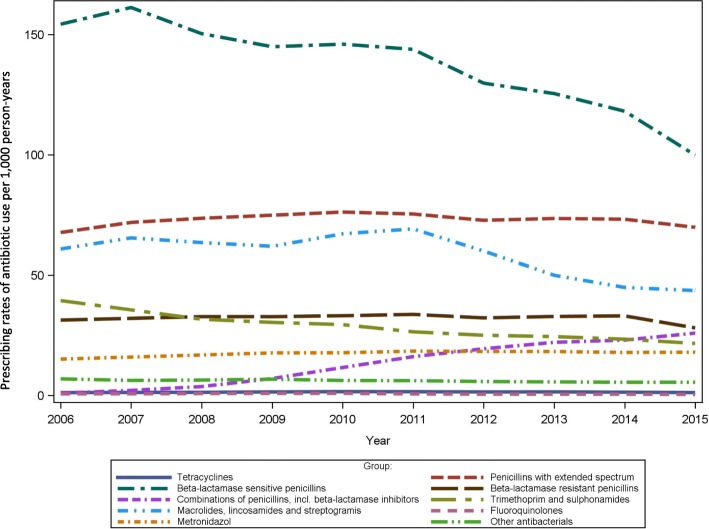
Prescribing rates of antibiotic subgroup use per 1000 person-years 2006 to 2015

The most frequently used antibiotics through all years were beta-lactamase sensitive penicillins (J01 CE), and penicillins with extended spectrum (J01CA) (Fig. 6). The use of penicillins with extended spectrum (J01CA), sulfonamides and trimethoprim (J01E), fluoroquinolones (J01MA), as well as other anti-bacterials (J01X) was rather stable through the years across sex and age groups (Additional file 1: Figure S7). Use of sulfonamides and trimethoprim (J01E) was most frequent among adults above 85 years (123/1000 person-years) while the prescribing rates accounted for less than 41/1000 person-years in other age groups. A decrease in prescribing rates was observed for beta lactamase sensitive penicillins (J01 CE) for all patient groups. A decrease was also seen from 2011 to 2015, for the use of macrolides, lincosamides and streptogramins (J01F). The prescribing rates of beta-lactamase resistant penicillins (J01CF) was stable until 2014 after which a decrease was observed among all groups. A small increase was seen for use of metronidazol (G01AF01, J01XD01, P01AB01), especially for females, whereas a notably large increase in prescribing rates was found for combinations of penicillins including beta-lactamase inhibitors (J01CR). This increase was seen for both females and males and within all age groups throughout the years, but especially noted for persons above 65 years (Additional file 1: Figure S7). Similar trends were seen for the volume of subgroup antibiotics used (Additional file 1: Figure S8).

Differences in the age and sex standardized volume of different antibiotics were also observed between the municipalities (Additional file 1: Table S4). For instance, tetracycline (J01AA) use varied up to 4 times between different municipalities. In addition, use of macrolides (J01F) and fluoroquinolones (J01MA) was twice as high in some municipalities versus others. There was no major variation between municipalities in the use of combinations of penicillins including beta-lactamase inhibitors (J01CR).

Mean duration of antibiotic treatment in days varied between the antibiotic subgroups as expected, but also between age groups (Table 1). Adults over 85 years had 11 days and 14 days longer duration of antibiotic treatment with tetracyclines (J01AA) and sulfonamides/trimethoprim (J01E) respectively, compared to the youngest age group. In contrast, the youngest age group had 23 days longer duration of treatment with flourquinolones (J01MA) compared to the oldest individuals. For combinations of penicillins including beta-lactamase inhibitors (J01CR), a 2 day longer duration was observed for people below 65 years compared to people above 85 years. No variation in mean duration of antibiotic treatment was observed between females and males.

**Table 1:** Mean duration of antibiotic treatment in days according to sex and age groups in 2015

		Overall					Females					Males				
ATC codes	Antibiotic group	15–39	40–64	65–84	+ 85	Overall	15–39	40–64	65–84	+ 85	Overall	15–39	40–64	65–84	+ 85	Overall
J01, G01AF01, P01AB01, J04A	Overall	9.4	11.1	12.8	14.3	11.5	9.1	10.7	12.5	14.5	11.1	9.9	11.7	13.2	14.0	12.0
J01AA	Tetracyclines	31.0	31.5	32.1	47.9	31.5	29.4	31.1	32.2	41.5	30.4	34.5	31.9	32.0	68.8	33.0
J01CA	Penicillins with extended spectrum	10.6	12.1	13.1	13.3	12.2	10.3	11.7	12.8	13.3	11.8	13.1	13.5	13.6	13.3	13.5
J01 CE	Beta-lactamase sensitive penicillins	9.6	9.8	10.2	10.3	9.9	9.6	9.7	10.1	10.4	9.8	9.7	10.0	10.3	10.3	10.0
J01CF	Beta-lactamase resistant penicillins	11.1	12.1	13.4	13.6	12.2	11.0	11.6	12.8	13.8	11.9	11.2	12.5	13.8	13.2	12.5
J01CR	Combinations of penicillins, incl. Beta-lactamase inhibitors	17.1	17.0	15.7	15.3	16.2	17.5	17.0	15.5	15.4	16.2	16.6	17.0	15.9	15.2	16.2
J01E	Trimethoprim and sulphonamides	3.7	7.9	13.1	16.9	11.6	3.4	6.6	12.4	16.9	10.6	11.7	14.3	14.5	17.0	15.0
J01F	Macrolides, lincosamides and streptogramins	7.0	9.9	10.6	9.9	9.1	7.1	9.9	10.6	9.8	9.1	6.9	10.0	10.6	10.1	9.0
J01MA	Fluoroquinolones	30.1	14.3	12.0	9.9	14.3	27.3	12.6	13.1	7.4	14.0	34.0	16.3	11.3	13.0	14.5
G01AF01, J01XD01, P01AB01	Metronidazol	4.9	5.5	6.0	6.2	5.4	4.8	5.4	5.8	6.1	5.2	5.5	5.8	6.2	6.3	5.9
Other J01 codes	Other antibacterials	18.2	18.0	18.3	19.6	18.5	16.4	16.8	17.5	19.5	17.7	27.2	21.9	20.0	20.0	20.7

## Discussion

Prescribing rates of antibiotics have decreased considerably in Central Denmark after 2011 after an initial slight increase between 2006 and 2011. The decline in use began later (from 2013) and was less pronounced in the older age groups, whereas a similar decline was seen for both sexes. However, the pattern of much higher antibiotic use in females versus males, and among older versus younger people has remained also in the most recent years. The overall use of narrow-spectrum antibiotics decreased substantially over the years, while the prescribing rates of broad-spectrum antibiotic use first declined from 2011 onwards. Of concern, among females aged ≥65 years and males aged ≥85 years a continuously increasing trend in broad-spectrum antibiotic use was observed from 2006 to 2015, primarily related to increasing use of combinations of penicillins including beta-lactamase inhibitors. There was a clear decline in antibiotic use in all municipalities over time, although striking geographical differences remained in antibiotic use in 2015 that were not explained by age and sex differences.

### Comparison with other studies

Our results regarding the notably high antibiotic use among older persons match those observed in earlier studies from Denmark and United States, [8, 26, 27]. Community physicians may tend to treat older persons more aggressively with antibiotics and at a lower threshold, due to multiple comorbid conditions that may increase risk of adverse outcomes from untreated infections in older individuals. On the other hand, a firm medical indication for antibiotics should preferably be present in older persons before treatment, due to increased risk of side effects of antibiotics in older people including *Clostridium difficile* colitis, interactions with other medications in those with polypharmacy, or risk of treatment-induced cognitive disturbances [28–30].

The overall prescribing rates of antibiotic use were relatively stable from 2006 to 2011, however when we examined prescribing rates according to narrow- and broad spectrum antibiotics a different picture was seen (Fig. 4), as the prescribing rates of narrow-spectrum antibiotics decreased while the rates of broad-spectrum antibiotics increased at the same time. This is in accordance with a previous Dutch study [12], which argue that some doctors may have used broad-spectrum antibiotics to replace narrow-spectrum antibiotics. In contrast to the Dutch study, the use of broad-spectrum antibiotics decreased from 2011 to 2015 in our population. This could be related to a national antibiotic campaign from 2013 to 2015, which focused on a more prudent choice of antimicrobials when antibiotics is necessary emphasizing the importance of using older narrow spectrum drugs including beta-lactamase sensitive penicillins as first choice. However, still the decrease in broad-spectrum antibiotics was much lower than that for narrow-spectrum antibiotics, primarily related to the large increase in use of combinations of penicillins including beta-lactamase inhibitors. In our latest study year, we therefore observed that for the first time in Denmark, prescribing of broad-spectrum antibiotics surpassed prescribing of narrow-spectrum antibiotics.

The increase in use of broader spectrum antibiotics is worrying as their use is related to resistance and adverse drug events including gastrointestinal disturbances, secondary infections (including yeast and C difficile infections), nephrotoxicity, neurological or psychiatric effects, sensory or motor disturbances, and allergic reactions [31]. Occurrence of such antibiotic-associated adverse events is a threat to patient safety and costly for society [32]**.**

Within the broad-spectrum antibiotics, a large increase was observed for the combination penicillins with beta-lactamase inhibitors, in particular among older people. One could speculate that this may be related to changes in regional and national recommendations for treatment of moderate exacerbations in patients with chronic obstructive pulmonary disease (COPD) in 2007, where amoxicillin with clavulanic acid became a first line drug in Denmark instead of narrow-spectrum penicillins [33]. Denmark has one of the highest prevalence of COPD worldwide [34], and patients with COPD and respiratory infections are primarily seen at general practitioners. The experienced effectiveness of amoxicillin with clavulanic acid likely has made this antibiotic popular in all middle-aged and older adults with respiratory infections, of whom many have comorbidities, not only in COPD patients. A new review has found that clinicians’ fear of complications from infections may be a reason for prescribing broader spectrum agents than necessary [31].

### Strengths and limitations

A major strength of this study is the use of complete individual-level prescription drugs bought at monopolized community pharmacies. All antimicrobial agents in Denmark can be bought as prescription medication only and redeemed prescriptions will therefore be a truthful reflection of the actual utilization out of hospitals. Our study also has some limitations. The study was restricted to Central Denmark Region, but the homogeneity of the Danish Healthcare System makes it likely that our results can be generalized to the four other regions in Denmark [22]. Moreover, we were not able to directly link the use of antibiotic agents to potential underlying diagnoses in the study population. It is a limitation that we in this drug utilization study are not able to make firm conclusions about the potential causality between the national stewardship program and the changes over time in antibiotic use. It would in this context also be relevant to examine the possible impact of changing prescription behavior on the antibiotic resistance. Unfortunately, we did not have the possibility to analyse the occurrence of individual level antibiotic resistance among people with recent antibiotic exposure. However, the national stewardship program was the most important specific intervention focused on utilization of antibiotics in the Danish healthcare system during the study period. No substantial changes in the overall antibiotic resistance rates were reported in this period by the Danish Ministry of Health (Statens Serum Institut), which also supports the hypothesis that the stewardship may be an important factor in the changing pattern of antibiotic use. In addition, the Danish healthcare system has otherwise been quite homogeneous and stable throughout the period and no major demographic changes occurred in the population except for some ageing, which we accounted for in the analyses.

### Perspective

Our data show that there is room for improvement in antibiotic prescribing. The decrease in overall antibiotic use mainly stems from a decrease in narrow-spectrum antibiotics, which in general should be the preferred antibiotics due to lower risk of transmissible resistance development in bacteria. The very large increase observed for use of combinations of penicillins with beta-lactamase inhibitors is worrying and tailored interventions for reducing the use is needed. Furthermore, there remains huge variation between Danish municipalities in the use of critical antibiotics including tetracyclines, macrolides and fluoroquinolones. The fast trends in use of individual antibiotics and the huge variation between Danish municipalities may suggest that the use of antibiotics could be moved by safety concerns or market factors including pricing, availability and advertising rather than current guidelines [35, 36]. Increased focus on avoiding unnecessary use of combinations of penicillins with beta-lactamase inhibitors, tetracyclines, macrolides and fluoroquinolones may be required in Denmark.

## Conclusions

This study provides evidence for substantially decreasing prescribing rates for antibiotics in Central Denmark after 2011. At the same time, the use of broad-spectrum antibiotics has increased and now for the first time surpasses the use of narrow-spectrum antibiotics in Denmark.

## Additional file

Additional file 1: **Table S2**. shows volume of antibiotics DDD per 1000 person-years from 2006 to 2015 according to sex and age groups. **Table S3** shows volume of narrow- and broad-spectrum antibiotics DDD per 1000 person-years from 2006 to 2015, stratified by sex and age group and **Table S4** shows age and sex standardized volume of subgroup of antibiotics, stratified by the municipalities in Central Denmark Region in 2015. Furthermore the file contains **Figures S7** and **S8**, which shows prescribing rates and volume of antibiotic per 1000 person-years from 2006 to 2015, stratified by sex and age group. (DOCX 1211 kb)

## Abbreviations

ATC: Anatomical Therapeutic Chemical
COPD: Chronic obstructive pulmonary disease
DDD: Defined Daily Doses
