# Supplementary material for: Decreasing trends, and geographical variation in outpatient antibiotic use: a population-based study in Central Denmark
Source: BMC Infect Dis. 2019 Apr 24;19:337. doi: 10.1186/s12879-019-3964-9 (PMC6480614; doi:10.1186/s12879-019-3964-9)
Supplement: Supplementary file 1 — Table S2. shows volume of antibiotics DDD per 1000 person-years from 2006 to 2015 according to sex and age groups. Table S3 shows volume of narrow- and broad-spectrum antibiotics DDD per 1000 person-years from 2006 to 2015, stratified by sex and age group and Table S4 shows age and sex standardized volume of subgroup of antibiotics, stratified by the municipalities in Central Denmark Region in 2015. Furthermore the file contains Figures S7 and S8, which shows prescribing rates and volume of antibiotic per 1000 person-years from 2006 to 2015, stratified by sex and age group. (DOCX 1211 kb) [file 12879_2019_3964_MOESM1_ESM.docx]

**Additional file 1**

**Table 2: Volume of antibiotics DDD per 1,000 person-years from 2006 to 2015 according to sex and age groups**

|  | **Overall** | | | | |  | **Female** | | | | |  | **Male** | | | | |
| --- | --- | --- | --- | --- | --- | --- | --- | --- | --- | --- | --- | --- | --- | --- | --- | --- | --- |
| **År** | **15-39** | **40-64** | **65-84** | **+85** | **Overall** |  | **15-39** | **40-64** | **65-84** | **+85** | **Overall** |  | **15-39** | **40-64** | **65-84** | **+85** | **Overall** |
| **2006** | 3,837.7 | 4,286.5 | 8,178.3 | 18,979.6 | 5,041.9 |  | 4,936.7 | 5,092.8 | 8,906.1 | 20,640.2 | 6,141.3 |  | 2,776.8 | 3,498.2 | 7,312.4 | 15,367.2 | 3,920.5 |
| **2007** | 4,051.1 | 4,551.3 | 8,509.7 | 20,268.7 | 5,333.4 |  | 5,213.6 | 5,427.4 | 9,254.6 | 21,933.2 | 6,497.9 |  | 2,929.4 | 3,695.4 | 7,635.5 | 16,616.9 | 4,147.7 |
| **2008** | 3,876.5 | 4,508.0 | 8,414.5 | 21,438.6 | 5,271.0 |  | 5,057.8 | 5,324.8 | 9,256.4 | 23,239.8 | 6,447.8 |  | 2,739.2 | 3,710.2 | 7,439.2 | 17,485.1 | 4,075.8 |
| **2009** | 3,816.4 | 4,491.6 | 8,737.1 | 22,213.1 | 5,328.6 |  | 5,016.4 | 5,313.4 | 9,684.5 | 24,156.5 | 6,544.0 |  | 2,659.7 | 3,687.8 | 7,653.1 | 17,983.8 | 4,094.6 |
| **2010** | 3,946.9 | 4,696.6 | 8,907.9 | 22,095.8 | 5,518.7 |  | 5,201.5 | 5,568.4 | 9,866.7 | 24,004.4 | 6,772.6 |  | 2,735.8 | 3,841.7 | 7,825.3 | 17,946.5 | 4,245.0 |
| **2011** | 3,964.1 | 4,749.1 | 8,955.7 | 22,205.7 | 5,588.8 |  | 5,258.1 | 5,660.7 | 9,997.9 | 23,980.7 | 6,881.5 |  | 2,714.6 | 3,853.6 | 7,795.2 | 18,399.4 | 4,276.7 |
| **2012** | 3,581.4 | 4,470.6 | 8,970.6 | 22,724.7 | 5,374.5 |  | 4,829.9 | 5,322.0 | 10,004.5 | 24,269.5 | 6,623.4 |  | 2,379.7 | 3,633.5 | 7,829.0 | 19,499.4 | 4,109.4 |
| **2013** | 3,249.7 | 4,321.0 | 9,016.8 | 23,432.6 | 5,236.6 |  | 4,442.9 | 5,155.8 | 10,050.7 | 25,151.5 | 6,470.5 |  | 2,103.9 | 3,497.9 | 7,883.2 | 19,889.0 | 3,987.6 |
| **2014** | 3,175.2 | 4,174.2 | 8,722.4 | 23,042.1 | 5,113.4 |  | 4,343.3 | 4,997.8 | 9,714.5 | 25,239.6 | 6,332.6 |  | 2,057.3 | 3,359.9 | 7,639.3 | 18,664.2 | 3,880.3 |
| **2015** | 2,741.4 | 3,802.8 | 8,240.7 | 22,332.2 | 4,709.1 |  | 3,806.0 | 4,626.0 | 9,242.9 | 24,439.4 | 5,883.3 |  | 1,726.9 | 2,988.7 | 7,152.3 | 18,233.1 | 3,524.5 |

**Table 3: Volume of narrow- and broad-spectrum antibiotics DDD per 1,000 person-years from 2006 to 2015, stratified by sex and age group**

|  |  |  | **Overall** | | | | |  | **Female** | | | | |  | **Male** | | | | |
| --- | --- | --- | --- | --- | --- | --- | --- | --- | --- | --- | --- | --- | --- | --- | --- | --- | --- | --- | --- |
|  |  |  |  | | | | |  |  | | | | |  |  | | | | |
| **Broad-spectrum**  **antibiotics** | **År** |  | **15-39** | **40-64** | **65-84** | **+85** | **Overall** |  | **15-39** | **40-64** | **65-84** | **+85** | **Overall** |  | **15-39** | **40-64** | **65-84** | **+85** | **Overall** |
|  | 2006 |  | 1,038.8 | 1,419.0 | 3,210.6 | 5,937.0 | 1,647.9 |  | 1,548.2 | 1,824.4 | 3,516.6 | 6,199.2 | 2,133.6 |  | 547.0 | 1,022.7 | 2,846.6 | 5,366.6 | 1,152.3 |
|  | 2007 |  | 1,135.8 | 1,561.1 | 3,501.1 | 6,322.9 | 1,807.5 |  | 1,687.0 | 2,030.4 | 3,860.1 | 6,550.6 | 2,346.8 |  | 604.1 | 1,102.6 | 3,079.7 | 5,823.2 | 1,258.3 |
|  | 2008 |  | 1,168.4 | 1,582.2 | 3,564.1 | 6,723.0 | 1,855.1 |  | 1,774.2 | 2,048.7 | 3,950.2 | 6,961.2 | 2,421.2 |  | 585.2 | 1,126.6 | 3,116.8 | 6,200.2 | 1,280.0 |
|  | 2009 |  | 1,188.9 | 1,647.4 | 3,828.2 | 7,258.4 | 1,954.6 |  | 1,811.5 | 2,143.3 | 4,266.2 | 7,390.5 | 2,549.2 |  | 588.8 | 1,162.5 | 3,327.1 | 6,970.9 | 1,350.9 |
|  | 2010 |  | 1,275.6 | 1,833.0 | 4,128.9 | 7,689.3 | 2,139.8 |  | 1,925.1 | 2,377.9 | 4,624.6 | 7,878.0 | 2,778.9 |  | 648.7 | 1,298.7 | 3,569.2 | 7,279.0 | 1,490.7 |
|  | 2011 |  | 1,344.3 | 1,953.7 | 4,364.0 | 8,262.9 | 2,287.0 |  | 2,029.3 | 2,547.7 | 4,914.8 | 8,355.9 | 2,969.0 |  | 682.8 | 1,370.1 | 3,750.6 | 8,063.4 | 1,594.7 |
|  | 2012 |  | 1,246.9 | 1,895.5 | 4,483.7 | 8,863.0 | 2,279.2 |  | 1,931.8 | 2,477.0 | 5,068.8 | 8,961.9 | 2,967.9 |  | 587.7 | 1,323.8 | 3,837.6 | 8,656.5 | 1,581.5 |
|  | 2013 |  | 1,202.0 | 1,873.4 | 4,611.4 | 9,609.2 | 2,308.8 |  | 1,886.8 | 2,477.2 | 5,203.9 | 9,763.5 | 3,015.4 |  | 544.4 | 1,277.9 | 3,961.7 | 9,290.9 | 1,593.4 |
|  | 2014 |  | 1,205.5 | 1,821.6 | 4,440.8 | 9,628.1 | 2,273.3 |  | 1,894.8 | 2,442.3 | 5,057.6 | 10,065.1 | 2,999.2 |  | 545.7 | 1,207.9 | 3,767.5 | 8,757.4 | 1,539.2 |
| **Narrow-spectrum**  **antibiotics** | 2015 |  | 1,139.0 | 1,769.5 | 4,385.4 | 9,584.9 | 2,226.6 |  | 1,787.8 | 2,364.2 | 5,016.8 | 9,959.4 | 2,926.9 |  | 520.8 | 1,181.4 | 3,699.6 | 8,856.2 | 1,520.2 |
|  | 2006 |  | 2,402.1 | 2,364.3 | 2,773.0 | 3,641.6 | 2,471.6 |  | 2,790.5 | 2,556.1 | 2,703.9 | 3,405.3 | 2,697.4 |  | 2,027.2 | 2,176.8 | 2,855.1 | 4,155.6 | 2,241.3 |
|  | 2007 |  | 2,516.0 | 2,480.5 | 2,893.1 | 3,961.7 | 2,593.1 |  | 2,934.2 | 2,695.4 | 2,806.8 | 3,783.9 | 2,839.2 |  | 2,112.6 | 2,270.6 | 2,994.4 | 4,351.9 | 2,342.5 |
|  | 2008 |  | 2,318.2 | 2,403.6 | 2,802.8 | 3,943.3 | 2,469.1 |  | 2,715.2 | 2,559.1 | 2,747.4 | 3,709.1 | 2,686.5 |  | 1,936.0 | 2,251.7 | 2,866.9 | 4,457.5 | 2,248.3 |
|  | 2009 |  | 2,198.5 | 2,307.4 | 2,760.1 | 3,949.8 | 2,376.6 |  | 2,588.4 | 2,458.0 | 2,705.2 | 3,740.7 | 2,590.5 |  | 1,822.7 | 2,160.2 | 2,822.9 | 4,405.0 | 2,159.4 |
|  | 2010 |  | 2,252.2 | 2,327.4 | 2,712.2 | 3,796.5 | 2,397.1 |  | 2,666.6 | 2,476.0 | 2,652.3 | 3,552.8 | 2,613.7 |  | 1,852.2 | 2,181.7 | 2,779.9 | 4,326.3 | 2,177.1 |
|  | 2011 |  | 2,226.1 | 2,272.4 | 2,626.7 | 3,679.9 | 2,349.4 |  | 2,653.2 | 2,416.1 | 2,587.7 | 3,531.4 | 2,572.7 |  | 1,813.6 | 2,131.1 | 2,670.1 | 3,998.3 | 2,122.7 |
|  | 2012 |  | 1,962.1 | 2,045.3 | 2,482.3 | 3,624.6 | 2,129.7 |  | 2,339.4 | 2,144.6 | 2,401.3 | 3,450.5 | 2,307.9 |  | 1,599.0 | 1,947.7 | 2,571.8 | 3,988.0 | 1,949.3 |
|  | 2013 |  | 1,688.0 | 1,927.9 | 2,440.8 | 3,655.9 | 1,972.2 |  | 2,008.4 | 1,996.6 | 2,348.6 | 3,494.9 | 2,116.7 |  | 1,380.4 | 1,860.1 | 2,541.8 | 3,987.7 | 1,825.9 |
|  | 2014 |  | 1,655.7 | 1,832.3 | 2,296.2 | 3,424.9 | 1,891.6 |  | 1,958.9 | 1,871.2 | 2,152.4 | 3,284.6 | 2,004.5 |  | 1,365.5 | 1,793.9 | 2,453.1 | 3,704.5 | 1,777.3 |
|  | 2015 |  | 1,317.6 | 1,524.6 | 1,913.3 | 2,897.1 | 1,554.4 |  | 1,561.8 | 1,566.4 | 1,808.4 | 2,799.4 | 1,652.7 |  | 1,084.8 | 1,483.2 | 2,027.1 | 3,087.1 | 1,455.1 |

**Figure 7: Prescribing rates of antibiotic subgroup DDD per 1,000 person-years from 2006 to 2015, stratified by sex and age group**

**
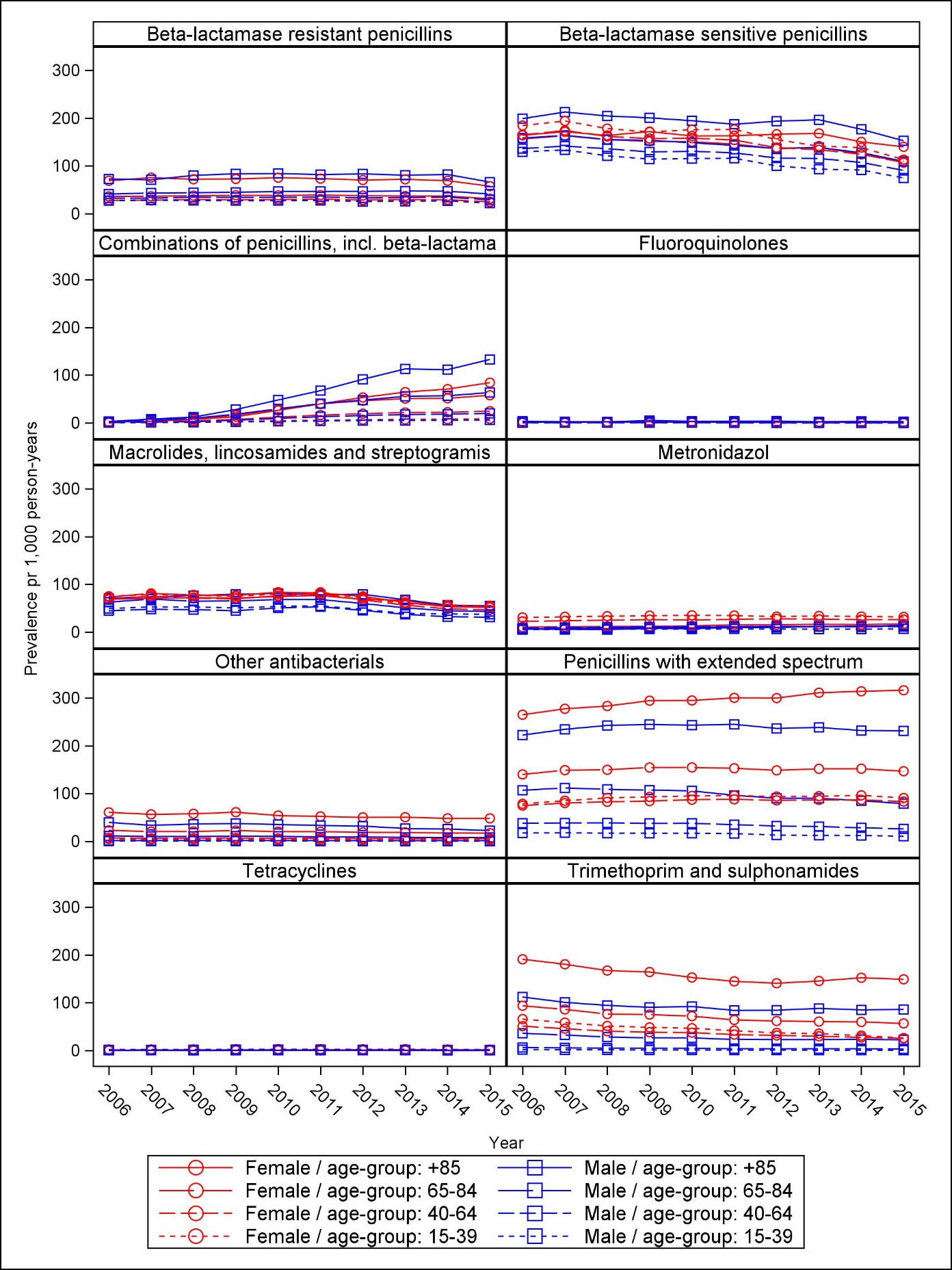
**

Prescribing rates of antibiotic use per 1,000 person-years

**Figure 8: Volume of antibiotic subgroup DDD per 1,000 person-years from 2006 to 2015, stratified by sex and age group**

**
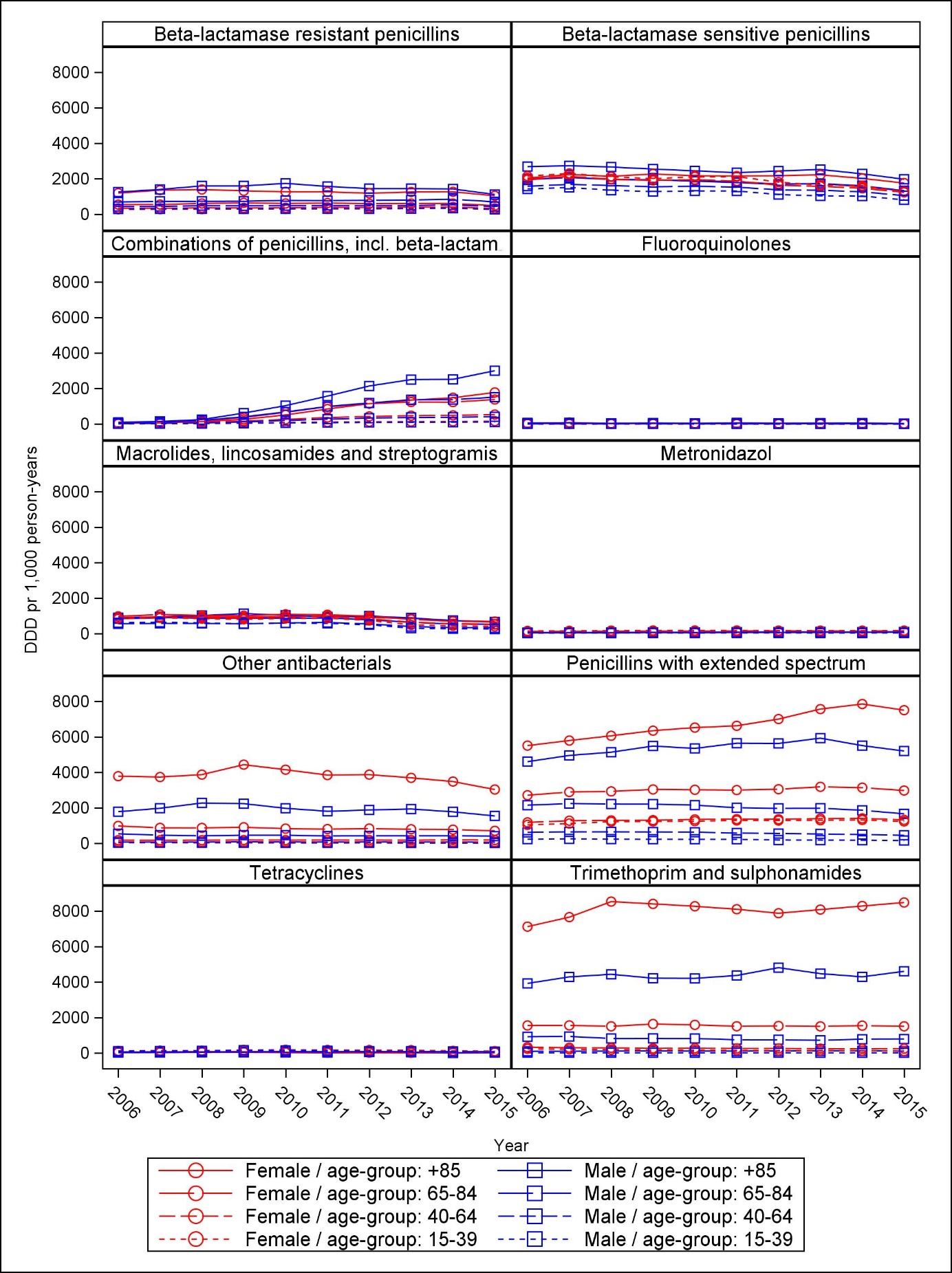
**

**Table 4: Age and sex standardized volume of subgroup of antibiotics, stratified by the municipalities in Central Denmark Region in 2015**

| **Group** | **Favrskov** | **Hedensted** | **Herning** | **Holstebro** | **Horsens** | **Ikast-**  **Brande** | **Lemvig** | **Norddjurs** | **Odder** | **Randers** | **Ringkøbing-**  **Skjern** | **Samsø** | **Silkeborg** | **Skander-**  **borg** | **Skive** | **Struer** | **Syddjurs** | **Viborg** | **Århus** |
| --- | --- | --- | --- | --- | --- | --- | --- | --- | --- | --- | --- | --- | --- | --- | --- | --- | --- | --- | --- |
| Overall | 4,428.1 | 4,983.3 | 4,872.0 | 4,679.9 | 4,963.4 | 4,788.7 | 5,073.4 | 5,210.7 | 4,193.9 | 4,790.3 | 4,655.1 | 3,724.5 | 4,534.1 | 4,584.3 | 5,604.8 | 5,237.0 | 4,478.7 | 4,677.1 | 4,602.8 |
| Tetracyclines | 63.0 | 48.1 | 113.5 | 55.2 | 83.2 | 155.6 | 60.4 | 107.8 | 48.3 | 34.7 | 87.6 | 0.0 | 62.1 | 42.0 | 41.3 | 55.6 | 123.9 | 62.4 | 56.0 |
| Penicillins with extended spectrum | 1,326.1 | 1,097.6 | 1,252.5 | 1,221.6 | 1,240.7 | 1,311.6 | 1,365.9 | 1,301.9 | 904.3 | 1,278.5 | 1,018.8 | 635.7 | 1,273.2 | 1,224.0 | 1,607.3 | 1,224.1 | 1,097.1 | 1,247.2 | 1,244.4 |
| Beta-lactamase sensitive penicillins | 1,091.4 | 1,354.1 | 1,090.3 | 1,219.8 | 1,285.4 | 1,185.1 | 1,334.7 | 1,164.0 | 1,238.7 | 1,250.7 | 1,250.1 | 944.9 | 1,049.8 | 1,213.1 | 1,270.0 | 1,244.4 | 1,130.4 | 1,128.8 | 1,087.4 |
| Beta-lactamase resistant penicillins | 431.4 | 409.1 | 390.1 | 379.0 | 363.5 | 370.0 | 438.9 | 482.4 | 400.9 | 371.8 | 385.7 | 473.1 | 413.3 | 407.4 | 496.4 | 433.2 | 428.3 | 432.1 | 384.0 |
| Combinations of penicillins, incl. beta-lactamase inhibitors | 579.1 | 618.7 | 507.0 | 484.4 | 628.3 | 474.2 | 528.7 | 633.6 | 550.4 | 671.4 | 458.1 | 832.2 | 556.2 | 525.3 | 614.2 | 463.5 | 595.0 | 614.3 | 572.4 |
| Trimethoprim and sulphonamides | 340.2 | 431.9 | 802.2 | 560.3 | 404.2 | 659.1 | 509.6 | 531.1 | 346.7 | 383.6 | 647.0 | 132.2 | 483.7 | 463.7 | 633.2 | 903.9 | 358.2 | 339.5 | 463.1 |
| Macrolides, lincosamides and streptogramis | 350.4 | 591.9 | 393.3 | 476.6 | 508.1 | 323.0 | 464.4 | 428.4 | 302.1 | 397.2 | 461.9 | 305.9 | 357.0 | 315.8 | 648.9 | 652.1 | 367.4 | 489.0 | 403.1 |
| Fluoroquinolones | 11.0 | 6.6 | 3.5 | 5.6 | 8.6 | 7.5 | 8.7 | 5.6 | 1.8 | 4.6 | 6.3 | 5.0 | 4.7 | 5.9 | 5.0 | 6.4 | 7.2 | 5.6 | 5.0 |
| Metronidazol | 106.4 | 97.3 | 92.5 | 107.9 | 89.5 | 84.7 | 99.9 | 128.1 | 102.5 | 150.8 | 76.9 | 79.2 | 125.7 | 106.8 | 98.4 | 112.4 | 92.9 | 109.6 | 107.9 |
| Other antibacterials | 113.1 | 307.4 | 210.9 | 136.1 | 330.0 | 207.6 | 255.2 | 393.4 | 246.7 | 220.5 | 223.7 | 306.9 | 188.2 | 274.7 | 180.4 | 118.1 | 273.3 | 235.6 | 263.8 |
